# Associations of Problematic Smartphone Use and Smartphone Screen Time With Eating Disorder Psychopathology in Non-Clinical Samples: A Systematic Review

**DOI:** 10.2196/88572

**Published:** 2026-03-09

**Authors:** Johanna Keeler, Laura Conde Ludtke, Qingyu Yang, Valentina Raschke Rameh, Rebecca Ward, Janet Treasure, Ben Carter

**Affiliations:** 1 Centre for Research in Eating and Weight Disorders, Department of Psychological Medicine, Institute of Psychiatry, Psychology and Neuroscience, King's College London, 16 De Crespigny Park, London, SE5 8AF, United Kingdom, +44 (0)207 848 0071; 2 Department of Child and Adolescent Psychiatry, Psychosomatics and Psychotherapy, LVR University Hospital Essen, University of Duisburg-Essen, Essen, Germany; 3 Department of Psychosis Studies, Institute of Psychiatry, Psychology and Neuroscience, King's College London, London, United Kingdom; 4 South London and Maudsley NHS Foundation Trust, London, United Kingdom; 5 Department of Biostatistics and Health Informatics, Institute of Psychiatry, Psychology and Neuroscience, King's College London, London, United Kingdom

**Keywords:** eating disorders, disordered eating, problematic smartphone use, smartphone addiction, systematic review., mobile phone

## Abstract

**Background:**

The ubiquitous use of smartphones has given rise to maladaptive patterns of use, often termed “problematic smartphone use” (PSU), which disproportionately impacts children and young people and is associated with poor mental health. Emerging evidence suggests that patterns of smartphone use (eg, PSU and high smartphone screen time) may also influence eating patterns and contribute to symptoms associated with eating disorders (ED), although the nature of this relationship remains poorly understood.

**Objective:**

The aim of this systematic review was to examine the association between PSU and ED psychopathology or ED-related outcomes (eg, body dissatisfaction, emotional eating, and food addiction) in clinical and nonclinical populations and explore potential moderators and mediators.

**Methods:**

This preregistered systematic review conducted according to PRISMA (Preferred Reporting Items for Systematic Reviews and Meta-Analyses) guidelines searched 3 databases (PubMed, Embase, and Web of Science) for studies published after January 2011 reporting data on PSU and ED psychopathology.

**Results:**

Thirty-five studies met the prespecified eligibility criteria, with almost all reporting cross-sectional data in nonclinical populations (n=52,584; mean age 17.0, SD 5.5 years). Most studies were assessed as being of good quality (n=28, 78%) according to a modified version of the Newcastle-Ottawa Scale. In these nonclinical samples, the vast majority of studies reported a positive association between PSU and ED psychopathology, which was largely consistent across age groups and countries. Identified mediators of this relationship included greater emotional regulation difficulties and anxious and depressive symptoms. Positive associations were also found across studies between PSU and several ED-related outcomes including food addiction, body dissatisfaction, uncontrolled eating, and emotional overeating. Daily smartphone screen time was consistently related to higher ED psychopathology. According to a GRADE (Grading of Recommendations, Assessment, Development, and Evaluation) assessment of the evidence, most outcomes were rated as low certainty, largely due to the cross-sectional nature of evidence, which contributed to a high risk of bias.

**Conclusions:**

PSU and greater daily smartphone screen time are associated with higher ED symptoms, body image dissatisfaction, and broader disordered eating behaviors. Due to a paucity of studies in clinical populations, these conclusions are generalizable only to nonclinical populations (ie, those without a formal diagnosis of an ED). Further longitudinal research in clinical populations is needed to fully contextualize the impact of PSU and smartphone screen time on ED risk and severity.

## Introduction

Eating disorders (EDs) encompass a group of psychiatric conditions characterized by persistent disturbances in eating behaviors that can result in significant physical, psychological, and social morbidity [1]. According to the *DSM-5* (*Diagnostic and Statistical Manual of Mental Disorders* [Fifth Edition]), the primary diagnostic categories include Anorexia Nervosa, Bulimia Nervosa, Binge Eating Disorder, Other Specified Feeding and Eating Disorder, and Avoidant/Restrictive Food Intake Disorder [2], although between these diagnostic categories, there are often similarities in symptom presentation (eg, binge eating and food restriction). The typical onset of EDs is in adolescence or young adulthood, and estimates of lifetime risk range from 8.4% (3.3%‐18.6%) for women and 2.2% (0.8%‐6.5%) for men, with atypical EDs such as Other Specified Feeding and Eating Disorder and binge-type EDs such as Binge Eating Disorder and Bulimia Nervosa being more common [3]. However, these figures likely underestimate the true burden since many individuals with subclinical or “prodromal” ED symptoms do not seek help [4]. For example, studies have estimated that 22% of adolescents [5] and 31% of adults [6] experience some form of disordered eating.

Across modern theoretical models, there is a consensus that EDs have complex origins including sociocultural, biological, psychological, and environmental factors [7-11], some of which are modifiable targets for intervention. Estimates of recovery are variable depending on the type of ED, although outcomes improve substantially when the illness is identified and treatment is provided early [12]. This underscores the importance of identifying modifiable risk factors for both clinical and subclinical ED symptoms to develop effective prevention programs and early intervention strategies [13]. Factors associated with smartphones such as excessive usage and nocturnal screen exposure may constitute such modifiable risk factors [14].

The use of smartphones is ubiquitous, having become deeply integrated into daily life, and has fundamentally changed the way people communicate, work, and engage with society [15]. There is debate over the role of smartphones in society as there are clear benefits in terms of social connectedness and productivity, but there is also a growing scientific literature detailing its harms to health [16,17]. The exposure of individuals to harm from smartphones has been operationalized in 2 ways, by a crude measure of the amount of time the devices are used (herein smartphone screen time) and by maladaptive use mirroring a behavioral addiction, described as problematic smartphone use (PSU).

In brief, PSU refers to a behavioral or psychological dependence on smartphones. This encompasses the inability to regulate or control smartphone use, preoccupation with the smartphone, and use of smartphones in inappropriate situations such as when driving an automobile, which has detrimental effects on daily living. PSU is distinguished from normal smartphone usage, or broader measures of smartphone use such as screen time, by symptoms such as severe cravings or withdrawal symptoms (eg, anger, restlessness, and anxiety) that interfere with functioning, as well as excessive use and difficulty self-regulating usage. Importantly, PSU has not yet been formally recognized in the *DSM-5* or the *ICD-11* (*International Classification of Diseases, Eleventh Revision*) [18,19], highlighting an ongoing debate over whether PSU reflects a distinct clinical diagnosis or a broader maladaptive smartphone behavior, although it has been argued that it can still operationally be considered a form of behavioral addiction [20]. PSU is, however, the preferred term used in this paper in order to avoid overpathologizing, as has been highlighted within this debate.

PSU is an increasing issue impacting individuals across the lifespan, although it disproportionately impacts adolescents and young adults, with approximately 1 in 4 young people experiencing PSU [20]. It has been linked to adverse mental health outcomes including higher rates of depressive and anxiety symptoms [20], as well as wider impacts on sleep quality [21] and feelings of loneliness via a reduction in face-to-face interaction [22]. Physical consequences of PSU include sedentary behavior, neck and shoulder pain, and reduced physical fitness [23-25]. In line with this, poorer dietary quality and higher levels of nutritional deficiencies have been found to be associated with PSU in students [26]. A high incidence of depressive and anxiety comorbidity [27], sleep problems [28,29], and feelings of loneliness and isolation [30] are all commonly seen in EDs, which may contribute to their onset and maintenance.

Although prior systematic reviews have examined problematic internet usage [31,32], social media exposure [33-43], and specific harmful online content [44,45] in relation to ED symptoms and related constructs such as body image and body dysmorphia, in various clinical and nonclinical populations, none have examined PSU specifically. Aspects of PSU may be more relevant to EDs, given that core behaviors in EDs (eg, calorie counting and overexercising) may be facilitated by smartphone apps such as calorie and fitness trackers, as well as social media apps that may facilitate bodily or lifestyle comparison. People with EDs often experience social isolation [30,46], which could also increase their reliance on smartphones or alter their relationship with their smartphone.

Through systematically collating the results of available studies, this study aims to investigate the association between PSU (or associated measures such as smartphone screen time) and symptoms associated with EDs. For completeness, we included outcome measures associated with ED psychopathology that are risk factors for the development of an ED (eg, body dissatisfaction and excessive exercise [47]) or overlap with ED presentations (eg, emotional eating and food addiction [48,49]). Secondary aims are to identify potential sources of heterogeneity such as moderators (eg, age group, and sex of the participants) as well as mediators of this association.

## Methods

This systematic review is reported according to the PRISMA (Preferred Reporting Items for Systematic Reviews and Meta-Analyses) guidelines [50]. The study protocol was preregistered via PROSPERO on February 27, 2025 (registration number CRD420250654159).

### Search Strategy

A systematic search was conducted on September 26, 2025, independently by 3 reviewers (QY, LCL, and VR) across 3 electronic databases (PubMed, Embase, and Web of Science) for studies published from January 1, 2011 (after which research incorporating smartphones began to emerge [20,51]).

Databases were searched for papers including in their titles and abstracts terms, such as “cell phone” or “smartphone” or “app” in combination with “addiction” or “problematic behavior” or “dependence” and “eating disorder” or “disordered eating” or “body dissatisfaction” or “emotional eating” (see Table S1 in Multimedia Appendix 1 for a full description of the search terms used per database). The searches were supplemented by manual screening of reference lists from included studies and citation tracking via Google Scholar to identify additional eligible studies not retrieved in the initial search.

### Eligibility Criteria

Studies were included if they (1) examined the quantitative association between PSU and ED symptoms, using measures of effect size (eg, correlation coefficients and *ß* values), group differences (eg, *t* values and *F* values), and/or statistical significance (eg, *P* values); (2) assessed PSU using validated self-report measures that map onto at least 1 aspect of the criteria for behavioral addiction [20] (eg, Smartphone Addiction Scale [SAS] and Problematic Mobile Phone Use Questionnaire), or related indicators such as smartphone screen time (eg, hours per minutes of daily phone use); (3) assessed ED psychopathology or related symptoms using either validated self-report questionnaires (eg, Eating Disorder Examination-Questionnaire, Eating Disorder Inventory, Binge Eating Scale, Appearance Evaluation Scale, and Exercise Addiction Inventory) or structured diagnostic interviews (eg, SCID-5 and MINI); and (4) were published in English in peer-reviewed journals.

Studies were excluded if they (1) used PSU measures that were not specific to smartphone use, or that combined smartphone use with broader internet use or screen-based activity without reporting data separately; (2) were systematic reviews, scoping reviews, narrative reviews, meta-analyses, theses, commentaries, editorials, conference abstracts, or book chapters; (3) did not include validated measures for both PSU and ED symptoms; or (4) were published before January 1, 2011. Data from participants of any gender, age, or nationality were included.

### Data Screening and Extraction

Titles and abstracts were screened independently by 2 reviewers (QY and LCL) against basic eligibility criteria including language type, sample type, study focus, and paper type, and noneligible studies were excluded. Full-text papers of potentially eligible studies were then screened independently by the same 2 reviewers. Reasons for exclusion at the full-text stage were documented. Disagreements at any stage of the screening process were first discussed between the 2 reviewers, and unresolved cases were referred to the wider team (VRR, JLK, and BC).

Data extraction was carried out by 2 reviewers (QY and LCL) using a bespoke database and 50% of the extracted data were independently reviewed by another reviewer (JLK). Extracted variables included publication details (author, year), country, study design, setting, sample characteristics (eg, size, age, and gender), clinical characteristics (eg, ED diagnosis, BMI, and comorbidities), and the validated measures used to assess ED symptoms and PSU. Extracted data relating to results of the studies included a narrative summary of results, statistical results (eg, *P* values, *r* values, *ß* values, *F* values, and 95% CIs), covariates, and findings relating to studied moderators or mediators where applicable.

### Assessment of Study Quality and Certainty of Evidence

The quality assessment of included studies was carried out independently by 2 reviewers (QY and LCL) using a version of the Newcastle-Ottawa Scale (NOS) [52] modified for assessing cross-sectional studies and adapted to the topic of this review (Table S2 in Multimedia Appendix 1 [23,53-86]). The NOS is a widely used tool for assessing the quality of non–randomized observational studies according to 3 overarching categories: (1) the selection of the study groups, (2) the comparability of the groups, and (3) the ascertainment of the outcomes. Stars are awarded for each item, which determines the overall quality of the domain. Each study was then judged overall as good, fair, or poor quality. Studies were rated as “good” when all domains were rated good or only 1 was rated fair, with no poor ratings. They were rated as “fair” when 1 poor rating was present alongside otherwise fair or good domains, or when multiple domains were fair without any poor. Finally, studies were rated as “poor” when 2 or more domains were rated poor, or when a single poor rating was found in a critical domain such as outcome measure. Discrepancies were resolved by consensus and consultation with senior reviewers (JLK and BC).

 Following preregistration, the decision was made to include an assessment of the quality and certainty of evidence using the evidence-grading system developed by the GRADE (Grading of Recommendations, Assessment, Development, and Evaluation) collaboration (Table S3 in Multimedia Appendix 1) [87]. One senior author (JLK) initially applied the GRADE system, followed by an assessment of the evidence ratings for each outcome with another senior reviewer (BC). A final decision on the ratings was reached through discussion and consensus. As our review included only cross-sectional data (ie, observational studies), we downgraded the evidence for each outcome, starting from low quality, when there was a serious limitation in relation to a particular factor or by 2 levels if we considered that there was a very serious limitation. Evidence was only upgraded in the case of very large, consistent, and plausible associations, in line with GRADE guidance for observational studies. The GRADEpro (Evidence Prime) [88] software was used to determine the certainty of evidence and generate the Summary of Findings table (Table S3 in Multimedia Appendix 1).

### Data Synthesis

Due to the contextual heterogeneity of the included studies in terms of population characteristics, outcome measures, and different statistical reporting formats, a narrative synthesis was conducted to summarize the findings. To explore patterns across studies, results were organized into key themes according to ED outcome, with the primary focus being results relating to ED symptomatology, with other themes including body image and broader altered or disordered eating behaviors (eg, food addiction and emotional eating). Within these themes, results were synthesized according to statistical approach, including between-group comparisons, linear associations (regression models and correlations), and mediation and moderation analyses. Results were also narratively synthesized for measures of PSU and smartphone screen time separately. Study characteristics and a brief summary of the key results, including statistical notation where available and appropriate, are presented per individual study in a summary table. No data conversions or sensitivity analyses were performed.

## Results

### Search Results

**Figure 1.**
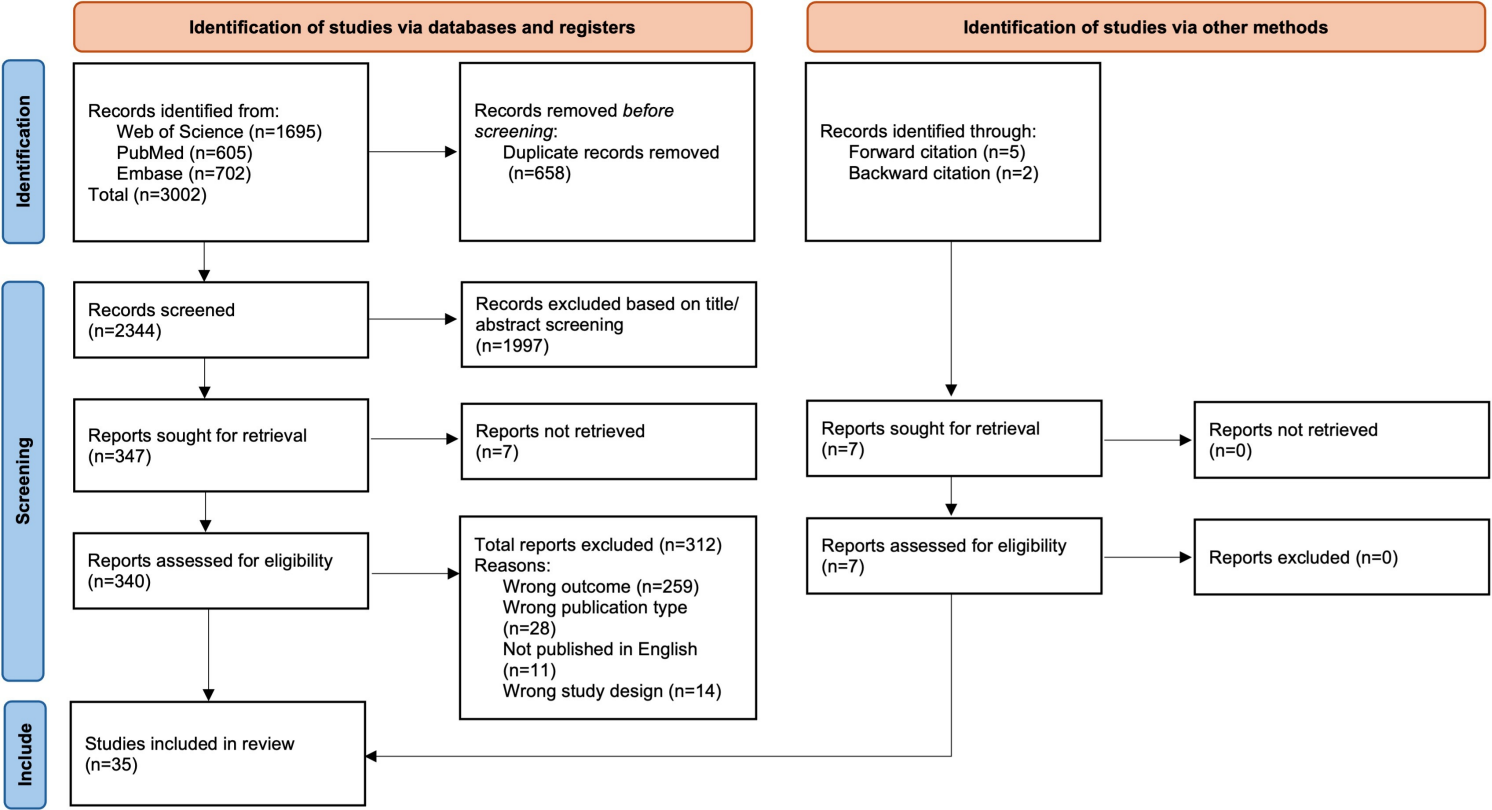
Flow diagram of included studies (adapted from Page et al [89], with permission).

A total of 3002 papers were identified, of which the full texts of 340 were assessed against the full eligibility criteria. Of these, 28 met the criteria for inclusion and an additional 7 studies were identified through backward and forward citation tracking, resulting in a total of 35 studies included in the review (Figure 1).

### Characteristics of Included Studies

Table 1 shows the methodological and sample characteristics of the included studies. The combined sample size across studies was 52,584, ranging from 86 [53] to 10,246 [54]. Studies were published between 2019 and 2025 and were conducted in Turkey (n=9), China (n=9), the United States (n=4), Germany (n=3), Spain (n=2), the United Arab Emirates (n=1), Bahrain (n=1), Italy (n=1), Austria (n=1), Brazil (n=1), South Korea (n=1), India (n=1), and Singapore (n=1). The vast majority of studies (n=32) was cross-sectional, apart from 3 studies that were prospective cohort, randomized controlled trials, and longitudinal studies, reporting cross-sectional data available for inclusion in this study.

**Table 1.** Study and sample characteristics of included studies (n=35)^a^.

Reference, country	Study design	Setting	Sample, N	Gender, (female), %	Age (years), mean (SD)	PSU^b^ measure	ED^c^ measure
Bernabé-Mateo et al (2025) [72], Spain	Cross-sectional	University (nursing students)	350	78.6	NR^d^, 19.9 (5.3)	NMP-Q^e^	YFAS^f^ 2.0
Chen et al (2025) [81], China	Cross-sectional	Junior high schools in southern China	808	50.7	12‐16 14.2 (0.9)	MPAI^g^	BAS-2^h^
Chu et al (2024) [54], United States	Prospective cohort reporting cross-sectional data	Community	10,246	48.6	9‐14, 9.9 (0.6)	MPIQ^i^	KSADS-5^j^
de Heselle and Montag (2024) [53], Germany	RCT^k^ with cross-sectional data	School and university	86	100	≥18, 24.0 (4.6)	SAS-SV^l^ Daily screen time (hour and minute per day)	BIAS-BD^m^; MBSRQ-AS^n^
Domoff et al (2020) [85], United States	Cross-sectional	Community	111	55.9	13‐16, 14.6 (1.1)	APU^o^	DEBQ^p^, YFAS-C^q^
Emirtekin et al (2019) [80], Turkey	Cross-sectional	High school	443	60	14‐18, 16.0 (1.1)	SAS-SV	BIDS^r^
Gokce and Ozer (2021) [67], Turkey	Cross-sectional	University	319	67.4	18‐33, 21.0 (2.1)	PU^s^	EAT^t^
Grant et al (2019) [65], United States	Cross-sectional	University	3425	64.2	NR (NR)	SAS-SV	BDD-Q^u^; MIDI^v^
Hasan et al (2023) [64], United Arab Emirates	Cross-sectional	University	552	79.5	NR, 21.2 (5.1)	SAS-SV	EAT-26
Jahrami et al (2021) [73], Bahrain	Cross-sectional	Community	654	54	18‐35, 27.2 (5.1)	NMP-Q	YFAS
Kardeş et al (2023) [57], Turkey	Cross-sectional	University	347	59.9	18‐32, 21.1 (2.0)	SAS	EAT-40
Li et al (2022) [23], China	Longitudinal study with cross-sectional data	University	1181	50.7	18‐22, 18.9 (0.9)	MPATS^w^	C-EDE-QS^x^
Li et al (2025) [76], China	Cross-sectional	University (nursing students)	437	82.2	NR, 19.2 (0.9)	MPAI	DEBQ
Liu et al (2020) [78], China	Cross-sectional	High school	1036	44.4	11‐15, 12.4 (0.7)	SAS	BDS^y^
Liu et al (2023) [79], China	Cross-sectional	University	5909	53.8	18‐32, 19.9 (1.7)	SAS-SV	BPSS^z^
Lo Coco et al (2022) [82], Italy	Cross-sectional	Middle and high school	647	56.7	NR, 14.2 (1.4)	SPAI-I^aa^	BES^ab^
Ma et al (2025) [69], China	Cross-sectional	High school and college	9270	48.3	16 (med, IQR: 14‐19)	SAS-SV	SCOFF^ac^
Mayerhofer et al (2024) [63], Austria	Cross-sectional	School and university	913	82.4	14‐20 (med, IQR: 17, 15‐18)	SAS-SV Daily smartphone use (hours per day)	SCOFF
Miranda et al (2021) [84], Brazil	Cross-sectional	School	405	100	14‐19, 15.9 (1.3)	Smartphone screen time (minute per day)	BSQ^ad^
Örnek and Gündoğmuş (2022) [60], Turkey	Cross-sectional	University	358	59.8	NR, 22.3 (3.1)	SAS-SV	EAT-40
Park et al (2022) [71], South Korea	Cross-sectional	School	209	55.5	NR, 12.9 (0.7)	Korean Smartphone Overdependence Scale for Adolescents	YFAS-C; CEBQ^ae^
Pekgör and Eryılmaz (2021) [55], Turkey	Cross-sectional	Hospital clinic	113	85.6	18‐65 (med, IQR: 35, 31‐45)	SAS-SV	YFAS; CEBQ
Peris et al (2020) [83]*,* Spain	Cross-sectional	School	447	56.2	13‐16, 14.9 (0.8)	ERA-RSI^af^	BSS^ag^
Piko et al (2022) [58], India	Cross-sectional	High school	112	47.3	14‐18, 16.0 (1.1)	SAS-SV	EAT-26
Rozgonjuk et al (2023) [70], Germany	Cross-sectional	Community	119	100	18‐49, 23.1 (4.6)	Daily screen time (hour per day)	EDE-Q^ah^
Sanlier et al (2024) [62], Turkey	Cross-sectional	Community	643	62.7	18‐50, 26.5 (9.6)	SAS	EAT-26
Sezer et al (2025) [74], Turkey	Cross-sectional	School	437	79.9	15‐26, 16.3 (1.2)	SAS-SV	TFEQ^ai^
Tayhan and Yabancı (2021) [59], Turkey	Cross-sectional	University	437	73.5	19‐29, 20.7 (1.6)	SAS Daily smartphone screen time (hour per day)	EAT-40
Türkkan et al (2025) [75], Turkey	Cross-sectional	Community	604	82.9	18‐45, 21.1 (3.0)	SAS-SV	TFEQ
Wang et al (2023) [61], China	Cross-sectional	University	1112	61.1	17‐29, (21.4 (3.2)	MPAI Daily smartphone screen time (hour per day)	EAT-26
Watkins et al (2025) [56], United States	Cross-sectional	School	45	51.0	11‐14, 13.1 (1.3)	Daily screen time (hour day)	BSQ, BAS-2
Wickord and Quaiser-Pohl (2022) [68], Germany	Cross-sectional	Community	398	78.2	14‐67, 25.9 (11.1)	MPPUS-27^aj^	ISR^ak^
Wu et al [2021] [66], China	Cross-sectional	University	4325	61.4	NR, 19.9 (1.3)	SAS-SV	EAT-26
Yang et al (2022) [77], China	Cross-sectional	University	5986	54.1	17‐32, 19.8 (1.75)	SAS-SV	SAS DEBQ
Yang et al (2020) [86], Singapore	Cross-sectional	Community	100	100	13‐18,15.1 (1.3)	Daily smartphone screen time (hour per day)	BES-21

a The main findings of the included studies can be found in Table S4 in Multimedia Appendix 1 [23,53-86].

b PSU: problematic smartphone use.

c ED: eating disorder.

d NR: not reported.

e NMP-Q: Nomophobia Questionnaire.

f YFAS: Yale Food Addiction Scale.

g MPAI: Mobile Phone Addiction Index.

h BAS-2: Body Appreciation Scale—2.

i MPIQ: Mobile Phone Involvement Questionnaire.

j KSADS-5: Kiddie Schedule for Affective Disorders and Schizophrenia.

k RCT: randomized controlled trial.

l SAS-SV: Smartphone Addiction Scale—Short Version.

m BIAS-BD: Body Image Assessment Scale-Body Dimension.

n MBSRQ-AS: Multidimensional Body-Self Relations Questionnaire Appearance Scale.

o APU: Addictive Patterns of Use Scale.

p DEBQ: Dutch Eating Behavior Questionnaire.

q YFAS-C: Yale Food Addiction Scale for Children.

r BIDS: Body Image Dissatisfaction Scale.

s PU: Problematic Mobile Phone Use Scale.

t EAT: Eating Attitudes Test.

u BDD-Q: Body Dysmorphic Disorder Questionnaire.

v MIDI: Minnesota Impulse Disorder Interview.

w MPATS: Mobile Phone Addiction Tendency Scale.

x C-EDE-QS: Chinese version of Short Form of the Eating Disorder Examination Questionnaire.

y BDS: Body Dissatisfaction Scale.

z BPSS: The Satisfaction and Dissatisfaction with Body Parts Scale.

aa SPAI-I: Italian version of the Smartphone Addiction Inventory.

ab BES: Body Esteem Scale.

ac SCOFF: Sick Control One Fat Food.

ad BSQ: Body Shape Questionnaire.

ae CEBQ: Child Eating Behavior Questionnaire.

af ERA-RSI: Scale of Risk of Addiction to Social Media and the Internet for Adolescents.

ag BSS: Body Self-esteem Scale.

ah EDE-Q: Eating Disorder Examination-Questionnaire.

ai TFEQ: Three-Factor Eating Questionnaire.

aj MPPUS-27: Mobile Phone Problematic Use Scale.

ak ISR: ICD-10 Symptom Rating Scale.

Most samples were recruited from nonclinical settings, including universities (n=13), schools (n=11), both schools and universities (n=2), and the community (n=8). One study recruited the sample from a family medicine hospital polyclinic for weight loss [55]. Of the studies reporting age ranges (n=26), 11 studies were conducted in adolescent populations only (ie, those aged 18 years and younger) and 11 in adults only, of which 6 were in young adults (aged 18-35 years). Four studies included both adolescents and adults. The pooled mean (SD) age across studies was 17.0 (5.5) (reported by n=31 studies). Four studies were conducted in female-only samples, and in the rest, the percentage of female participants ranged from 44.4% to 85.6%.

In terms of the assessment of ED and ED-related symptoms, the most commonly used instrument was the Eating Attitudes Test (EAT [90]; n=9), used in its 26-item version in 5 studies, 40-item version in 3, and in 1 study the version was unclear. Other measures of ED symptoms included the Eating Disorder Examination Questionnaire (EDE-Q [91]; n=1) and in its 12-item form (Chinese version of Short Form of the Eating Disorder Examination Questionnaire [92]; n=1), and diagnostic or screening tools included the *ICD-10* (*International Statistical Classification of Diseases, Tenth Revision*) Symptom Rating Scale (ICD-10 Symptom Rating Scale [ISR] [93]; n=1), the Minnesota Impulse Disorders Interview [94], the Kiddie Schedule for Affective Disorders and Schizophrenia-5 (n=1 [95]), and the Sick, Control, One Stone, Fat, Food Questionnaire (SCOFF [96]; n=2). In terms of measures of relevant wider eating behaviors, the Yale Food Addiction Scale [97] was used in 5 studies, with a version for children (Yale Food Addiction Scale for Children [98]) in 2 studies. The Dutch Eating Behavior Questionnaire [99] was used in 3 studies and the Three-Factor Eating Questionnaire (TFEQ) [100] in 2 studies. Body image dissatisfaction was measured in 12 studies, using a variety of measures (Table 1).

The most common measure of PSU was the SAS (n=4 [101]) and its short form (SAS-SV; n=14 [102]). Three studies used the Mobile Phone Addiction Index [103], 2 used the Nomophobia Questionnaire [104], and other questionnaires assessing PSU used in single studies included the Mobile Phone Involvement Questionnaire [105], Addictive Patterns of Use Scale [106], Problematic Mobile Phone Use Scale, Mobile Phone Addiction Tendency Scale [107], Smartphone Addiction Inventory—Italian version [108], Scale of Risk of Addiction to Social Media and the Internet for Adolescents [109], Mobile Phone Problematic Use Scale [110] and the Korean Smartphone Overdependence Scale for Adolescents. Smartphone screen time was measured in 8 studies. All of the aforementioned instruments addressed at least 1 domain of PSU aligning with the *DSM-5* criteria for a behavioral addiction characterized by smartphone use.

### Quality Assessment of Included Studies

Seven studies included samples that were deemed representative of the target population, whereas 18 studies included samples that were deemed somewhat representative of the average target population (eg, due to nonrandom sampling). Only 10 studies provided justification for their sample size. Most used a validated measurement tool for PSU to ascertain the exposure (n=31), and very few studies used a nonvalidated measurement tool (eg, self-reported smartphone use only) (n=4). In terms of comparability, most studies accounted for age as a confounding variable (n=23), and most also controlled for at least 1 additional potential confounding factor (eg, sex, BMI; n=28). All studies used a validated measurement tool for ED or ED-related outcomes, and almost all studies presented an appropriate and clearly described statistical test for the measurement of the association (n=34). Overall, most included studies were rated as good (n=28) and some were rated as fair (n=6), primarily due to not controlling for confounding variables (Table S2 in Multimedia Appendix 1). Two studies [55,56] received a rating of “poor” as they used a nonrepresentative sample and failed to account for any confounders to address potential sources of bias.

### Main Findings of Included Studies

The full results of the included studies together with their characteristics are shown in Table S4 in Multimedia Appendix 1. Results were synthesized according to the type of ED or ED-related outcome, stratified according to the analytical approach taken. A total of 16 studies measured ED psychopathology, 5 studies measured food addiction symptoms, 6 measured other eating behaviors, and 12 measured body image–related outcomes.

### ED Psychopathology and PSU Outcomes

#### Measures of PSU and Smartphone Addiction

Ten studies adopted a dichotomous approach to assessing the relationship between PSU and ED psychopathology. Three of these studies assessed PSU scores according to whether individuals met a threshold on variations of the EAT questionnaire, all finding that participants exceeding this threshold (ie, at high risk of exhibiting disordered eating attitudes) had higher smartphone addiction scores than those who did not meet this threshold [57-59]. The GRADE of evidence was rated as low certainty for this finding, largely due to the cross-sectional nature of the studies. The remaining 7 studies examined ED psychopathology depending on whether the individuals were classed as meeting the threshold for PSU or smartphone addiction or not. In 3 studies using variations of the EAT questionnaire, disordered eating attitude scores were higher in those classed as smartphone addicted [60-62]. In 1 study, the chances of being screened for having disordered eating with the SCOFF questionnaire were higher in those who exceeded the smartphone addiction threshold [63]. In another study, exhibiting PSU was associated with higher odds of scoring highly in several specific ED-related symptom categories, including fearing weight gain, self-worth tied to weight, engaging in compensatory behaviors to prevent weight gain, binge eating, and distress with binge eating [54]. Contrarily, 1 study found that higher PSU risk was not associated with higher ED risk [64] and another study found that PSU was not associated with higher odds of screening positive for Binge Eating Disorder specifically [65]. Again, the GRADE of evidence was rated as low certainty across these findings.

Nine studies examined linear associations between PSU measures and ED symptoms. Seven studies used different variants of the EAT and various measures of smartphone addiction or PSU, most (n=5) of which found a positive association whereby greater PSU was associated with greater disordered eating attitude scores [57,60-62,66]. One of these studies controlled for a number of additional variables including BMI, depression and anxiety scores, smartphone usage, difficulty falling asleep at night, and frequency of physical activity [61]. These findings were also consistent in 1 study including age, gender, weight group, school use, age of smartphone usage, and family income status as variables within a multiple regression model [60]. On the contrary, 2 of these 7 studies failed to find a linear association between PSU scores and scores on the EAT [64,67].

The remaining 2 studies examined the linear association between PSU and other measures of ED psychopathology (EDE-Q and ISR). One study found that problematic mobile phone usage positively correlated with EDE-Q scores at baseline and at a 1-year follow-up [23]. The same study found that baseline problematic mobile phone usage positively predicted ED symptoms at 1 year but not vice versa [23]. Another study found a positive correlation between PSU scores and ED symptoms as measured by the ISR [68]. Overall, 7 out of 9 studies overall found a positive relationship between PSU and ED psychopathology. Due to the cross-sectional and associative nature of these studies, the GRADE of evidence was rated as low certainty across these findings.

Four studies examined mediators [23,66,69] and moderators [68]. In 1 study, the relationship between problematic mobile phone usage and ED symptoms was mediated by lower resilience scores [23]. Another study found that the relationship between poor sleep quality and higher disordered eating behaviors was mediated by greater PSU scores [66] as well as a sequential mediation pathway with greater PSU scores and greater psychological distress (both depression and anxiety) [66]. PSU scores were also found to positively mediate the relationship between depression and a likely ED diagnosis as determined by the SCOFF, as well as the relationship between loneliness and a likely ED, and the relationship between anxiety and a likely ED [69]. These findings were consistent when analyses were stratified by gender [69]. Finally, 1 study found that the positive relationship between ED symptoms and PSU was apparent only in “digital immigrants” (ie, individuals older than 40 years) but not in “digital natives” (ie, individuals younger than 40 years) [68].

#### Measures of Daily Smartphone Screen Time

In terms of daily smartphone use, it was found that people scoring above 30 on the EAT-40 [59] and those with a history of an ED diagnosis [70] had higher daily smartphone screen time. One study examined ED risk across categories of daily smartphone usage, finding that the odds of being screened positive for disordered eating with the SCOFF was higher in people using the smartphone for 7‐8 hours per day and more than 8 hours per day, but not 3‐4 or 5‐6 hours per day, compared with a reference category of 2 hours per day [63].

 Across 3 studies investigating linear associations between ED symptoms and smartphone screen time, 2 studies found a positive association between smartphone screen time and EAT-40 scores [59,61] and 1 study found a positive association with EDE-Q global scores and all subscales other than the Eating Concern subscale [70].

To summarize, across studies using different analytical approaches, there appears to be a positive association between measures of PSU, as well as smartphone screen time, and ED psychopathology. Furthermore, PSU and smartphone screen time (particularly where usage was more than 7 hours per day) is greater in people scoring highly on measures of ED psychopathology and vice versa; studies largely found greater ED psychopathology in individuals meeting a threshold for PSU or smartphone addiction.

### Food Addiction and PSU Outcomes

Five studies assessed the association between PSU or PSU-related outcomes and food addiction scores as measured by the Yale Food Addiction Scale. One study found that South Korean adolescents with a high risk of PSU had greater food addiction scores than adolescents with a low risk of PSU [71]. On the contrary, a study in Turkish adults found no greater food addiction scores in people categorized as having a smartphone addiction compared with those without smartphone addiction, although this study received a rating of “poor” as per the modified NOS [55]. Additionally, 1 study found that food addiction scores were no different across groups stratified by presence, risk, or absence of nomophobia (the fear of being without a mobile phone) [72]. An additional study examining nomophobia found no linear association between nomophobia and food addiction scores before and after adjusting for age, sex, BMI, and insomnia [73]. Due to the cross-sectional nature of the studies and high suspicion of publication bias, the GRADE of evidence was rated as very low certainty for this outcome. Publication bias cannot be ruled out, as the majority of studies reporting positive associations had a combined sample size similar to the single study reporting no association, suggesting that studies with null findings may have been less likely to have been published.

Despite this, 3 of these studies, as well as 1 additional study, found that measures of smartphone addiction [55,85], “overdependence” [71], or nomophobia [72] were positively correlated with food addiction scores. Again, the grade of evidence for these findings was rated as very low certainty. In one of these studies, this was the case when adjusting for age, sex, BMI, and socioeconomic status [71], but in another, the association was not found when stratifying the analysis by gender [72]. One study examining mediators reported that the positive association between smartphone addiction and food addiction was mediated by greater emotion regulation difficulties [85].

### Other Eating Behaviors and PSU Outcomes

Six studies examined the relationship between PSU-related outcomes and other eating behaviors such as emotional eating, cognitive restraint, and uncontrolled eating, as measured by the TFEQ (n=2), Dutch Eating Behavior Questionnaire (n=4), or Child Eating Behavior Questionnaire (n=1).

Both studies using the TFEQ found a positive correlation between smartphone addiction scores and the subscales of the TFEQ, including uncontrolled eating, cognitive restriction, and emotional eating [74,75]. In the former study, these associations remained significant when adding social media addiction scores and digital game addiction scores to a multiple regression model [74]. A positive association between smartphone addiction and emotional eating was also found in 2 additional studies [76,77], and in another study, it was found that adolescents with a higher risk of PSU had greater emotional overeating scores than adolescents with a lower risk of PSU [71]. Smartphone addiction was also positively associated with restrained eating [76,77,85], dysregulated eating [85], and external eating [76,77]. Similar to the previous outcomes, the GRADE evidence for the correlative findings pertaining to the relationship between PSU scores and emotional eating, as well as restrictive eating, was rated as low in both instances.

Three studies explored mediators [75,77,85]. It was found that emotion regulation difficulties significantly mediated the association between addictive phone use and dysregulated eating, restrained eating, and food addiction in 1 study [85]. In another, the relationship between body dissatisfaction and restrained eating, emotional eating, and external eating was mediated by smartphone addiction scores in 3 separate mediation models [77]. In this study, sequential mediation effects were found with smartphone addiction scores and depression symptoms as sequential mediators. Additionally, 1 study found that the relationship between smartphone addiction and higher BMI was positively mediated by all subscales of the TFEQ (higher uncontrolled eating, cognitive restriction, and emotional eating scores) in 3 separate mediation models [75].

### Measure of Body Image and PSU Outcomes

#### Measures of PSU and Smartphone Addiction

Eleven studies examined the relationship between body image–related outcomes and outcomes relating to PSU. One study assessing body dysmorphic disorder found that the presence of PSU was not associated with higher odds of screening positive for body dysmorphic disorder [65].

Several other studies assessed linear associations between PSU and body image and body dissatisfaction more broadly. In 4 studies, measures of smartphone addiction [77-79] and PSU [80] were positively associated with body dissatisfaction. Similarly, in 2 additional studies, smartphone addiction was negatively associated with body appreciation [81] and body esteem [82]. In the latter study, this association was found in both girls and boys (sex did not moderate the effect), but a positive association between smartphone addiction and attribution (the evaluation attributed to others about one’s own body and appearance) was found only in girls [82]. On the contrary, one study did not find a positive correlation between nomophobia scores and body satisfaction scores [83], and another study failed to find an association between PSU scores and body image dissatisfaction, appearance evaluation, and body area satisfaction [53]. The GRADE of evidence was rated as low certainty across these findings.

Across studies, identified mediators of the association between body dissatisfaction and greater PSU or smartphone addiction scores included depression and social anxiety (independently [80]), intrusive imagery and fear of negative evaluation (independently and sequentially [78]), and a tendency to present a positive view of oneself online [79]. Additionally, 1 study found that lower body appreciation mediated the relationship between smartphone addiction and poorer intuitive eating [81].

#### Measures of Daily Smartphone Usage

Four studies assessed the relationship between body image variables and smartphone screen time. One study in adolescents found that those with higher smartphone screen time had higher odds of having high body distortion and dissatisfaction than those with adequate smartphone screen time [84]. Another study found that smartphone screen time, together with social media addiction scores and sex, positively predicted body shape preoccupation scores [56]. However, another study found that smartphone screen time was not linearly related to several variables, including body image dissatisfaction, appearance evaluation, orientation, and body area satisfaction [53].

One study explored mediators, finding that the relationship between excessive smartphone use (>4 hours per day) and poorer body esteem was sequentially mediated by greater cognitive internalization of an ideal body image, greater appearance comparison, followed by greater appearance anxiety [86]. However, this relationship was no longer significant when controlling for total social media screen time, suggesting that social media usage was a predominant contributor to these results.

## Discussion

### Principal Findings

This systematic review assessing the association between PSU and ED psychopathology included 35 studies, predominantly reporting cross-sectional data in nonclinical populations. The majority of studies were rated as good quality. Most samples were recruited from schools and universities, and approximately half were adolescents and half (mostly young) adults. Across the included studies, it was broadly found that individuals scoring highly on PSU measures scored more highly on measures of ED psychopathology, and there were weak-to-moderate positive linear associations found. These findings were largely consistent across age groups and countries. Additionally, higher daily smartphone use was consistently related to higher ED psychopathology, particularly where daily use exceeded 7 hours per day [63]. Measures of PSU were also related to greater food addiction symptoms, broader disordered eating behaviors (eg, uncontrolled eating, emotional overeating, and cognitive restriction), and body dissatisfaction in most studies. The findings assessing the relationship between smartphone screen time and body dissatisfaction were more mixed. Across outcomes, a GRADE assessment of the evidence led to ratings of “low certainty” in most instances due to the cross-sectional nature of the evidence, apart from the outcomes relating to the association between PSU and food addiction, which received ratings of “very low certainty” largely due to substantially inconsistent findings across studies or potential publication bias. Longitudinal and interventional studies are needed to establish temporal and causal relationships.

The most consistently identified mediators of the relationship between PSU and ED psychopathology related to increases in negative affect, including emotion regulation difficulties and anxiety and depressive symptomatology. Maladaptive smartphone use may actively (or passively) increase one’s exposure to content related to thinness and body shaping, which may lead to negative affect via self-objectification [111] and increase the risk of developing ED symptoms [112]. In addition, night-time or late-evening smartphone use may interfere with sleep quality. Sleep problems are reported transdiagnostically across the ED spectrum [29] and in subclinical contexts are related to several eating behaviors such as impulsive eating and emotional eating [113]. Indeed, it was found that negative affect as a result of PSU acted as sequential mediators from poor sleep quality to higher ED behaviors in one study [66] and from greater body dissatisfaction to greater disordered eating behaviors in another [77]. Alternatively, individuals with ED symptoms may use smartphones to relieve or avoid negative emotions and stress [114], eventually developing a dependence on smartphones. However, it was found in 1 study that baseline PSU scores positively predicted ED symptoms at a 1-year follow-up but not vice versa [23], which, tentatively, could be a preliminary indicator of causality.

Traits common in individuals with, or at risk of developing, an ED, such as low self-esteem [115,116], perfectionism [117], features of addiction such as greater reward sensitivity, and impulsive traits such as negative urgency [118,119] and lack of premeditation [120] that are associated with PSU [121], may increase the risk of PSU or reliance on smartphones. Smartphones, with their ubiquitous use in everyday life, represent a convenient tool to consistently facilitate exposure to both content reflecting idealized depictions of lives and bodies and the opportunity to self-monitor one’s eating behaviors and exercise in a gamified and engaging way. In people with EDs, this convenience may be more pernicious, and the use of non–social media smartphone apps such as calorie-tracking and exercise-tracking apps [122,123], with reminders and gamified features (eg, streaks), may additionally increase an individual’s tendency to engage with their smartphone and reinforce dependency on these apps [124]. It is possible that this may also reinforce the addictive nature of some ED behaviors, such as body checking, compulsive exercising, binge eating, calorie counting, and restricting food intake. Moreover, individuals prone to developing disordered eating behaviors may exhibit an attentional bias toward ED-related content [125,126]. For these individuals, engagement with algorithms that learn, adapt, and are personalized via attention-related metrics may increase exposure to ED-related content [127] and promote body dissatisfaction via appearance and/or lifestyle comparison.

On the other hand, it is apparent from the results of this study that even for people without diagnosed EDs, PSU is associated with poorer body dissatisfaction and altered eating behaviors, which are likely to cause distress. During adolescence, individuals increasingly form their self-concept through observing others and their surroundings, making social comparison a mechanism for self-evaluation [128]. Smartphones provide a more convenient way for such comparisons. Adolescents with PSU may be frequently exposed to idealized appearance images, making them more likely to compare their own appearance with these “standards,” which leads to body dissatisfaction and negative self-evaluation. For young people and individuals at risk of developing an ED, or who are exhibiting prodromal symptoms of EDs, prevention programs and psychoeducation that incorporate media literacy may benefit from specifically including content relating to PSU. Early intervention pathways may benefit from incorporating psychoeducational content that targets the quality and nature of smartphone use, rather than focusing solely on reducing smartphone screen time. Additionally, emphasizing the benefits of engaging in more offline social interactions and activities that can help bolster one’s social connectedness and support, emotion regulation, and general psychological well-being may also enhance the quality of their interpersonal relationships, interpersonal confidence, and emotion regulation skills and reduce dependence on smartphones.

### Strengths and Limitations

To the authors’ knowledge, this review is the first to synthesize existing evidence on the relationship between PSU and EDs, as well as their related symptoms. The review adhered to a preregistered protocol and a comprehensive search was conducted across 3 databases, which was supplemented by manual screening of reference lists from included studies and citation tracking.

A major limitation pertaining to the included studies is that almost all included studies used cross-sectional designs. Given the cross-sectional nature of these data, it remains unclear whether PSU independently contributes to adverse psychological outcomes relating to ED symptoms, whether individuals with preexisting vulnerabilities are more likely to engage in PSU, or whether the relationship is bidirectional. Moreover, nearly all studies relied on self-report measures of both PSU and ED symptoms, which are subject to recall error and social desirability bias. In several studies, potential confounding variables such as gender, BMI, and age were considered; however, others that might influence the relationship between PSU and ED outcomes such as personality traits (eg, compulsivity and neuroticism [129]) and psychiatric comorbidity were not explored.

Such limitations underscore the need for future research using longitudinal or experimental designs, alongside objective measures and consideration of confounding factors, to strengthen the causal inference and reduce bias. As such, it is also important to acknowledge that ED outcomes were mostly assessed in nonclinical populations and utilized self-report questionnaires instead of formal diagnostic interviews, hospital admissions, or referrals to specialist services, meaning the findings cannot be fully generalized to clinical populations. Moreover, since patterns of social media overuse or maladaptive usage have been identified as plausible risk factors for the development of EDs cross-culturally [34], it is possible that problematic social media use contributed to our observed findings. Future studies should endeavor to simultaneously measure problematic social media usage and PSU, as well as specific app usage, to delineate pathways between these factors and specific ED symptoms and behaviors.

### Conclusions and Considerations for Future Research

The studies included in this systematic review indicated a significant and consistent association between PSU and ED symptoms and related outcomes such as food addiction, emotional overeating, and body dissatisfaction. The identification of key mediators such as emotion regulation difficulties, anxiety, and depression underscores the need to adopt a transdiagnostic approach in ED prevention and intervention. While these results align with existing literature demonstrating an association between PSU and negative mental health outcomes, the cross-sectional nature of the included studies limits causal inferences. Nevertheless, the findings of this review underscore the importance of integrating psychoeducation on PSU into ED prevention strategies and early intervention frameworks to enhance the timely detection and treatment of ED symptoms.

Crucially, future longitudinal and experimental research should seek to elucidate the bidirectional nature of this relationship, include individuals with and without clinically diagnosed EDs, and evaluate whether psychoeducational interventions targeting PSU can reduce the onset and severity of ED symptoms in at-risk populations. Patterns of use of specific apps that could contribute to or explicate the nature of PSU within ED populations should be explored, alongside a collation of qualitative evidence (eg, a qualitative metasynthesis) to enrich our findings.

## Supplementary material

10.2196/88572 Multimedia Appendix 1 Full quality assessment according to the Newcastle-Ottawa Scale (NOS) adapted for cross-sectional studies and Tables S1-S4.

10.2196/88572 Checklist 1 PRISMA (Preferred Reporting Items for Systematic Reviews and Meta-Analyses).

## Abbreviations:

*DSM-5*: *Diagnostic and Statistical Manual of Mental Disorders* (Fifth Edition)
EAT: Eating Attitudes Test
ED: eating disorder
EDE-Q: Eating Disorder Examination Questionnaire
GRADE: Grading of Recommendations, Assessment, Development, and Evaluation
*ICD-10*: *International Classification of Diseases, Tenth Revision*
*ICD-11*: *International Classification of Diseases, Eleventh Revision*
ISR: International Classification of Diseases-10 Symptom Rating Scale
NOS: Newcastle-Ottawa Scale
PRISMA: Preferred Reporting Items for Systematic Reviews and Meta-Analyses
PSU: problematic smartphone use
SAS: Smartphone Addiction Scale
SAS-SV: Smartphone Addiction Scale-Short Form
SCOFF: Sick, Control, One Stone, Fat, Food Questionnaire
TFEQ: Three-Factor Eating Questionnaire
