# Supplementary material for: Associations of Problematic Smartphone Use and Smartphone Screen Time With Eating Disorder Psychopathology in Non-Clinical Samples: A Systematic Review
Source: JMIR Ment Health. 2026 Mar 9;13:e88572. doi: 10.2196/88572 (PMC12980065; doi:10.2196/88572)
Supplement: Multimedia Appendix 1 [file mental-v13-e88572-s001.pdf]

Supplementary Material File for "Problematic smartphone use and smartphone screen time are associated with eating disorder psychopathology in non-clinical samples: a systematic review"

Table S1. Search terms utilised across databases (date limits: 01/01/2011 to 26/09/2025)

**PubMed**
(smartphone[tiab] OR smart phone[tiab] OR cellular phone[tiab] OR phone[tiab] OR cell phone[tiab] OR cell-phone[tiab] OR social media[tiab] OR iPhone[tiab] OR mobile device[tiab] OR mobile phone[tiab] OR iPad[tiab] OR Instagram[tiab] OR TikTok[tiab] OR Snapchat[tiab] OR Twitter[tiab] OR MyFitnessPal[tiab] OR screen time[tiab] OR app[tiab]) AND (problem* OR dependence OR dependency OR overuse OR addiction OR addictive OR nomophobia OR attachment OR excessive OR compulsive) AND (eating disord* [tiab] OR anorexi* [tiab] OR bulimi* [tiab] OR bing*[tiab] OR orthorexi*[tiab] OR body dysmorphia[tiab] OR night eating[tiab] OR disordered eating[tiab] OR purging[tiab] OR body image[tiab] OR eating behaviour[tiab] OR food addiction[tiab] OR body esteem[tiab] OR body dissatisfaction[tiab] OR body dysmorph* [tiab] OR dieting[tiab] OR OSFED[tiab] OR ARFID[tiab] OR bodyesteem[tiab] OR compulsive exercis*[tiab])
**Embase**
1. (mobile phone or smart phone or cell phone or Cellular Phone or phone or social media or iPhone or iPad or mobile device or Instagram or TikTok or Snapchat or Twitter or MyFitnessPal or screen time or app).ti,ab.<br>2. (problem* or dependenc* or overuse or addiction or addictive or nomophobia or attachment or excessive or compulsive).mp.<br>3. exp eating disorder/ or bing*.ti,ab. or orthorexi*.ti,ab. or disordered eat*.ti,ab. or purging.ti,ab. or exp body image/ or eating behaviour.ti,ab. or food addiction.ti,ab. or body esteem.ti,ab. or body dysmorph*.ti,ab. or dieting.ti,ab. or ARFID.ti,ab. or OSFED.ti,ab. or body-esteem.ti,ab. or compulsive exercis*.ti,ab. [mp=title, abstract, heading word, drug trade name, original title, device manufacturer, drug manufacturer, device trade name, keyword heading word, floating subheading word, candidate term word]<br>4. 1 and 2 and 3
**Web of Science**
1. ((((((((((((((AB=(smartphone)) OR AB=(smart phone)) OR AB=(cellular phone)) OR AB=(cell phone)) OR AB=(social media)) OR AB=(iPhone)) OR AB=(mobile device)) OR AB=(mobile phone)) OR AB=(iPad)) OR AB=(Instagram)) OR AB=(TikTok)) OR AB=(Snapchat)) OR AB=(Twitter)) OR AB=(MyFitnessPal)) OR AB=(screen time)) OR AB=(mobile app)<br>2. (((((((((AB=(problem)) OR AB=(dependence)) OR AB=(dependency)) OR AB=(overuse)) OR AB=(addiction)) OR AB=(addictive)) OR AB=(nomophobia)) OR AB=(attachment)) OR AB=(excessive)) OR AB=(compulsive)<br>3. ((((((((((((((((((AB=(eating disorder)) OR AB=(anorexia)) OR AB=(bulimia)) OR AB=(bing*)) OR AB=(orthorexia)) OR AB=(body dysmorphia)) OR AB=(night eating)) OR AB=(disordered eating)) OR AB=(purging)) OR AB=(body image)) OR AB=(eating behaviour)) OR AB=(food addiction)) OR AB=(body esteem)) OR AB=(body dissatisfaction)) OR AB=(dieting)) OR AB=(OSFED)) OR AB=(ARFID)) OR AB=(body-esteem)) OR AB=(compulsive exercise)<br>4. #3 AND #2 AND #1

1. Quality Assessment: Newcastle-Ottawa Scale (NOS) adapted for cross-sectional studies

This scale was adapted from the Newcastle-Ottawa Quality Assessment Scale for cohort studies to perform a quality assessment of cross-sectional studies for use in the current systematic review.

We have not selected one factor that is the most important for comparability, because the variables are not the same in each study. Thus, the principal factor should be identified for each study. We further adapted the scale from ascertainment of outcome (e.g. independent blind assessment/record linkage/self-report) to measurement of outcome to account for the methods used in this field of research.

Each study is evaluated against the criteria below and can be awarded up to 12 stars. The greater number of stars allocated indicates a higher quality.

Selection: (Maximum 6 stars)

1) Representativeness of the sample:
    a) Truly representative of the average in the target population (all subjects or random sampling) **
    b) Somewhat representative of the average in the target population (e.g., non-random sampling) *
    c) Selected group of users
    d) No description of the sampling strategy

2) Sample size:
    a) Justified and satisfactory * (A sample size >100 is satisfactory. If ≤100, it must be justified and meet the sample size calculation target)
    b) Not justified

3) Non-respondents:
    a) Comparability between respondents and non-respondent's characteristics is established, and the response rate is satisfactory. *
    b) The response rate is unsatisfactory, or the comparability between respondents and non-respondents is unsatisfactory.
    c) No description of the response rate or the characteristics of the responders and the non-responders.

4) Ascertainment of the exposure (Problematic smartphone use (PSU)):
    a) Validated measurement tool (e.g., Smartphone Addiction Scale (SAS) or objective usage data (e.g., hours/day) **
    b) Non-validated measurement tool, but the tool is available or described (e.g., self-reported smartphone use) *
    c) No description of the measurement tool

Domain scored: 0-1 (Poor), 2 (Fair), 3+ (Good) (Selection)

Comparability: (Maximum 3 stars)

1) The subjects in different outcome groups are comparable, based on the study design or analysis. Confounding factors are controlled.

a) The study controls for the most important factor (age) *
b) The study controls for an additional important factor (sex) *
c) The study controls for any additional factor (e.g., BMI, academic year, economic status, age of first smartphone use, GPA and household structure) *

Domain scored: 0 (Poor), 1 (Fair), 2+ (Good) (Comparability)

Outcome: (Maximum 3 stars)
1) Measurement of outcome (eating disorder psychopathology)

a) Validated measurement tool (e.g., EDE-Q, SCOFF, clinical interview) **
b) Non-validated measurement method, but the method is available or described (e.g., self-report) *
c) No description of the measurement tool

2) Statistical test:

a) The statistical test used to analyse the data is clearly described and appropriate, and the measurement of the association is presented, including confidence intervals and the probability level (*p*-value). *
b) The statistical test is not appropriate, not described or incomplete.

Domain scored: 0 (Poor); 1 (Fair); 2+ (Good) (Outcome)

The quality of each included study was evaluated based on the number of "good" and "fair" ratings it received according to the Newcastle-Ottawa Scale (NOS). Studies were rated as “good” when all domains were rated good or only one was rated fair, with no poor ratings. They were rated as “fair” when one poor rating was present alongside otherwise fair or good domains, or when multiple domains were fair without any poor. Finally, studies were rated as “poor” when two or more domains were rated poor, or when a single poor rating was found in a critical domain such as outcome measure.

Table S2. Newcastle Ottowa Scale assessment of study quality

Study reference	Selection (S)				Comparability (C)			Outcome (O)		Subtotal Assessment			
	Representativeness of the sample	Sample Size	Non-respondents	Ascertainment of the exposure	Most important confound controlled for (age)	Additional important confound controlled for 1 (sex)	Additional confound controlled for 2 (e.g., BMI)	Measurement of outcome	Statistical test	S[a]	C[b]	O[c]	Overall
Bernabé-Mateo et al., 2025	*	*		**			*	**	*	Good	Fair	Good	Good
Chen et al. 2025	*			**	*	*		**	*	Good	Good	Good	Good
Chu et al. 2024	**	*	*	**	*	*	*	**	*	Good	Good	Good	Good
de Heselle and Montag, 2024	*			**		*	*	**	*	Good	Good	Good	Good
Domoff et al. 2020	*			**		*		**	*	Good	Fair	Good	Good
Emirtekin et al. 2019	*			**	*	*		**	*	Good	Good	Good	Good
Gokce and Ozer, 2021	**	*		**				**	*	Good	Poor	Good	Fair
Grant et al. 2019	**			**				**	*	Good	Poor	Good	Fair
Hasan et al. 2023	*	*		**	*	*		**	*	Good	Good	Good	Good
Jahrami et al. 2021	*			**	*	*	*	**	*	Good	Good	Good	Good
Kardeş et al. 2023	*	*		**				**	*	Good	Poor	Good	Fair
Li et al. 2022	**	*		**	*	*	*	**	*	Good	Good	Good	Good
Li et al., 2025	*	*		**		*		**	*	Good	Fair	Good	Good
Liu et al. 2020	*			**	*	*		**	*	Good	Good	Good	Good
Liu et al. 2023	*			**	*	*	*	**	*	Good	Good	Good	Good
Lo Coco et al. 2022	*		*	**	*		*	**	*	Good	Good	Good	Good
Ma et al., 2025	*	*		**	*	*	*	**	*	Good	Good	Good	Good

Mayerhof et al. 2024	*			**	*	*	*	**	*	Good	Good	Good	Good
Miranda et al. 2021	**	*		*	*	*	*	**	*	Good	Good	Good	Good
Örnek and Gündoğmuş, 2022	*			**	*	*	*	**	*	Good	Good	Good	Good
Park et al. 2022	*			**	*	*	*	**	*	Good	Good	Good	Good
Pekgör and Eryılmaz, 2021				**				**		Fair	Poor	Good	Poor
Peris et al. 2020	**			**	*	*		**	*	Good	Good	Good	Good
Piko et al. 2022	*			**	*	*	*	**	*	Good	Good	Good	Good
Rozgonjuk et al. 2023	*	*		*		*		**	*	Good	Fair	Good	Good
Sanlier et al. 2024	*			**	*		*	**	*	Good	Good	Good	Good
Sezer et al. 2025	*	*		**	*	*	*	**	*	Good	Good	Good	Good
Tayhan and Yabancı, 2021	*	*		**				**	*	Good	Poor	Good	Fair
Türkkan et al. 2025	*	*		**				**	*	Good	Poor	Good	Fair
Wang et al. 2023	*	*		**				**	*	Good	Poor	Good	Fair
Watkins et al., 2025	*			*				**	*	Fair	Poor	Good	Poor
Wickord and Quaiser-Pohl, 2022	*			**	*	*		**	*	Good	Good	Good	Good
Wu et al. 2021	*			**	*	*	*	**	*	Good	Good	Good	Good
Yang et al. 2022	*			**	*	*	*	**	*	Good	Good	Good	Good
Yang et al. 2020	*			*	*	*	*	**	*	Fair	Good	Good	Good

Notes: Blank indicted the study did not meet the domain. Each domain was scored and rated as follows: [a]Domain scored: 0-1 (Poor), 2 (Fair), 3+ (Good) (Selection); [b]Domain scored: 0 (Poor), 1 (Fair), 2+ (Good) (Comparability); [c]Domain scored: 0 (Poor); 1 (Fair); 2+ (Good) (Outcome).

Table S3. GRADE assessment of the certainty of evidence

**Question:** Associations between eating disorder psychopathology and related outcomes and problematic smartphone usage (PSU) in non-clinical populations									
**№ of studies**	**Certainty assessment**						**Impact**	**Certainty**	**Importance**
	**Study design**	**Risk of bias**	**Inconsistency**	**Indirectness**	**Imprecision**	**Other considerations**			
**Higher PSU scores in individuals scored as high risk of exhibiting ED psychopathology vs. low risk of exhibiting ED psychopathology**									
3	non-randomised studies (cross-sectional)	serious	not serious	not serious	serious[a]	none	If the observed association reflects a true relationship, individuals from the general population (i.e., non-clinical) with a high risk of ED determined by ED psychopathology questionnaires may be more likely to display greater PSU.	⨁⨁◯◯ Low[a]	IMPORTANT
**Higher ED scores in individuals scored as high risk of PSU vs. low risk of PSU**									
7	non-randomised studies (cross-sectional)	serious	not serious	not serious	serious[b]	none	If the observed association reflects a true relationship, individuals from the general population (i.e., non-clinical) with a high risk of PSU may be more likely to display higher ED psychopathology.	⨁⨁◯◯ Low[b]	IMPORTANT
**Linear positive association between PSU scores and ED psychopathology**									
9	non-randomised studies (cross-sectional)	serious	not serious	not serious	serious[b]	none	If the observed linear association reflects a true relationship, higher PSU scores may be associated with higher ED psychopathology scores in the general (i.e., non-clinical) population.	⨁⨁◯◯ Low[b]	IMPORTANT
**Linear positive association between PSU scores and body image dissatisfaction**									
8	non-randomised studies (cross-sectional)	serious	not serious	not serious	serious[b]	none	If the observed linear association reflects a true relationship, higher PSU scores may be associated with higher body image dissatisfaction in the general (i.e., non-clinical) population.	⨁⨁◯◯ Low[b]	IMPORTANT
**Linear positive association between PSU scores and emotional eating**									
4	non-randomised studies (cross-sectional)	serious	not serious	not serious	serious[b]	none	If the observed linear association reflects a true relationship, higher PSU scores may be associated	⨁⨁◯◯ Low[b]	IMPORTANT

**Question:** Associations between eating disorder psychopathology and related outcomes and problematic smartphone usage (PSU) in non-clinical populations									
**№ of studies**	**Certainty assessment**						**Impact**	**Certainty**	**Importance**
	**Study design**	**Risk of bias**	**Inconsistency**	**Indirectness**	**Imprecision**	**Other considerations**			
							with higher levels of emotional eating in the general (i.e., non-clinical) population.		
**Linear positive association between PSU scores and restrained/restricted eating**									
5	non-randomised studies (cross-sectional)	serious	not serious	not serious	serious[b]	none	If the observed linear association reflects a true relationship, higher PSU scores may be associated with higher levels of restrained or restrictive eating in the general (i.e., non-clinical) population.	⨁⨁◯◯ Low[b]	IMPORTANT
**No difference in food addiction scores in individuals scored as high risk of PSU vs. low risk of PSU**									
3	non-randomised studies (cross-sectional)	serious	very serious[c]	not serious	very serious[d]	publication bias strongly suspected[e]	Individuals from the general population (i.e., non-clinical) with a high risk of PSU may not be more likely to display higher food addiction scores.	⨁◯◯◯ Very low[c,d,e]	NOT IMPORTANT
**Linear positive association between PSU scores and food addiction**									
5	non-randomised studies (cross-sectional)	serious	not serious	not serious	very serious[a]	publication bias strongly suspected[e,f]	If the observed linear association reflects a true relationship, higher PSU scores may be associated with higher levels of food addiction in the general (i.e., non-clinical) population.	⨁◯◯◯ Very low[a,e,f]	NOT IMPORTANT

Explanations:

a. Downgraded -1 due to moderate sample size (n = 500-1000) and consistency in direction of evidence, but absence of confidence intervals and inability to estimate the magnitude of association.

b. Downgraded -1 due to large total sample size (n > 1000) and overall consistency in direction of evidence, but absence of confidence intervals and inability to estimate the magnitude of association.

c. Inconsistency across studies in results.

d. Downgraded -2 due to moderate sample size (500-1000) but inconsistency in direction of evidence, absence of confidence intervals and inability to estimate the magnitude of the association.

e. One study assessed as poor quality.

f. One study had null findings with a large sample size equivalent to the combined sample size of the four studies that found positive associations.

Table S4. Study characteristics, sample characteristics and main findings of included studies (n = 35).

**Reference** ***Country***	**Study design**	**Setting**	**Sample N**	**Gender (% female)**	**Age range (M±SD)**	**PSU measure**	**ED measure**	**Comparisons (n)**	**Main findings**
Bernabé-Mateo et al., 2025 *Spain*	Cross-sectional	University (nursing students)	350	78.6%	NR (19.9±5.3)	NMP-Q	YFAS 2.0	Men (75) vs women (275)	- For both women and men, food addiction scores were no different between those with no nomophobia, at risk of nomophobia, and with nomophobia (*P*=.304 women; *P*=.057 men).<br>- However, regression analyses showed that greater food addiction scores were associated with greater nomophobia scores across the sample (β = 0.01; *P*=.002); but not for women and men separately (*P*=.119, .064, respectively).
Chen et al., 2025 *China*	Cross-sectional	Junior high schools in southern China	808	50.7%	12-16 (14.2±0.9)	MPAI	BAS-2	-	- Phone addiction was negatively associated with body appreciation (r = -0.55).<br>- (Lower) body appreciation mediated the relationship between (greater) phone addiction and (poorer) intuitive eating.
Chu et al., 2024 *USA*	Prospective cohort reporting cross-sectional data	Community	10,246	48.6%	9-14 (9.9±0.6)	MPIQ	KSADS-5	Problematic social media use (5,587) vs. problematic mobile phone use (7,280)	PSU associated with higher odds of:<br>- Fearing weight gain (OR = 1.43)<br>- Self-worth being tied to weight (OR = 1.51)<br>- Engaging in compensatory behaviours to prevent weight gain (OR = 1.26)<br>- Binge eating (OR = 1.66)<br>- Distress with binge eating (OR = 1.82)
de Heselle and Montag, 2024 *Germany*	RCT with cross-sectional data	School and university	86	100%	≥18 (24.0±4.6)	SAS-SV Daily screen time (h and min/day)	BIAS-BD; MBSRQ-AS	-	PSU and screen time showed no significant correlation with:<br>- Body image dissatisfaction<br>- Appearance evaluation<br>- Appearance orientation<br>- Body area satisfaction<br>- Overweight preoccupation<br>- Self-classified weight
Domoff et al., 2020 *USA*	Cross-sectional	Community	111	55.9%	13-16 (14.6±1.1)	APU	DEBQ, YFAS-C	-	Addictive phone use (APU) was positively associated with:<br>- Dysregulated eating (r = 0.53)<br>- Restrained eating (r = 0.39)<br>- Food addiction scores (r = 0.49)<br><br>Emotion regulation difficulties mediated the association between APU and:

									- Dysregulated eating<br>- Restrained eating<br>- Food addiction
Emirtekin et al., 2019<br>*Turkey*	Cross-sectional	High school	443	60%	14-18 (16.0±1.1)	SAS-SV	BIDS	-	- PSU was associated with body image dissatisfaction (ß = 0.11).<br>- Depression and social anxiety independently mediated the relationship between body image dissatisfaction and PSU.
Gokce and Ozer., 2021<br>*Turkey*	Cross-sectional	University	319	67.4%	18-33 (21.0±2.1)	PU	EAT	-	- Problematic mobile phone usage was not associated with EAT scores (r = 0.09) nor the presence of an eating behaviour disorder (*P* =.212).
Grant et al., 2019<br>*USA*	Cross-sectional	University	3,425	64.2%	NR (NR)	SAS-SV	BDD-Q; MIDI	PSU (687) vs. no PSU (2,738)	- PSU was not associated with higher odds of screening positive for BED (OR = 1.32; *P*=.283) or BDD (OR = 1.86; *P*.028; not significant after Bonferroni correction).
Hasan et al., 2023<br>*UAE*	Cross-sectional	University	552	79.5%	NR (21.2±5.1)	SAS-SV	EAT-26	Smartphone addiction risk (310) vs. no smartphone addiction risk (242)	- Risk of an eating disorder was not higher in those with a greater smartphone addiction risk (*p*=.550).<br>- Smartphone addiction score was linearly and negatively associated with EAT-26 scores when controlling for sleep quality and chronotype (B = -0.13) and when additionally controlling for age and sex.
Jahrami et al., 2021<br>*Bahrain*	Cross-sectional	Community	654	54%	18-35 (27.2±5.1)	NMP-Q	YFAS	-	- Food addiction scores were not significantly correlated with nomophobia.<br>- When adjusting for age, sex, BMI and insomnia, there remained no significant association between food addiction scores and nomophobia (ß = 0.20; *P*=.840).
Kardeş et al., 2023<br>*Turkey*	Cross-sectional	University	347	59.9%	18-32 (21.1±2.0)	SAS	EAT-40	-	- Smartphone addiction scores were higher in individuals who reported abnormal eating attitudes compared to those who did not report abnormal eating attitudes (*p* =.038).<br>- There was a positive association between smartphone addiction scores and EAT-40 scores (r = 0.14)
Li et al., 2022<br>*China*	Longitudinal study with cross-sectional data	University	1,181	50.7%	18-22 (18.9±0.9)	MPATS	C-EDE-QS	-	- Problematic mobile phone usage scores were positively correlated with C-EDE-QS scores at T1 (baseline; r = 0.36) and T2 (1y FU; r = 0.33).<br>- Problematic mobile phone usage scores at T1 positively predicted eating disorder symptoms at T2, but not vice versa.<br>- (Lower) resilience scores mediated the relationship between problematic mobile phone usage scores and eating disorder symptoms.

Li et al., 2025<br>*China*	Cross-sectional	University (nursing students)	437	82.2%	NR (19.2±0.9)	MPAI	DEBQ	-	- Mobile phone addiction was positively correlated with restrained eating (r = 0.24; *P*<.001), emotional eating (r = 0.23; *P*<.001) and external eating (r = 0.44; *P*<.001).<br>- A latent profile analysis identified high and low mobile phone addiction groups; the high addiction group had higher eating behaviour scores (*t* = -6.21; *P*<.001).
Liu et al., 2020<br>*China*	Cross-sectional	High school	1,036	44.4%	11-15 (12.4±0.7)	SAS	BDS	-	- Body dissatisfaction was positively correlated with smartphone addiction (r = 0.23; *P*<.001)<br>- The relationship between body dissatisfaction and smartphone addiction was mediated by (greater) intrusive imagery.<br>- (Greater) fear of negative evaluation was also a significant mediator.<br>- Intrusive imagery and fear of negative evaluation sequentially indirectly mediated the association between body dissatisfaction and smartphone addiction.
Liu et al., 2023<br>*China*	Cross-sectional	University	5,909	53.8%	18-32 (19.9±1.7)	SAS-SV	BPSS	-	- Body dissatisfaction was positively associated with smartphone addiction (r = 0.35)<br>- This association was mediated by (greater) tendency to present a positive view of themselves online
Lo Coco et al., 2022<br>*Italy*	Cross-sectional	Middle and high school	647	56.7%	NR (14.2±1.4)	SPAI-I	BES	Boys (280) vs. girls (367)	- Smartphone addiction scores were negatively associated with body esteem appearance and weight scores in both girls and boys, and with attribution scores in girls only.<br>- This remained the same when controlling for age and BMI.
Ma et al., 2025<br>*China*	Cross-sectional	High school and college	9,270	48.3%	16 (med, IQR: 14-19)	SAS-SV	SCOFF	-	- 41.4% of the sample met the screening threshold for possibly experiencing an eating disorder.<br>- Smartphone addiction scores positively mediated the relationship between: depression and (possible) EDs, loneliness and (possible) EDs, and social anxiety and (possible) EDs.<br>- The results were consistent when stratified by gender (male, female) and age group (11-15, 16-20, 21-25).
Mayerhof et al., 2024<br>*Austria*	Cross-sectional	School and university	913	82.4%	14-20 (med, IQR: 17, 15-18)	SAS-SV<br>Daily smartphone use (h/day)	SCOFF	PSU (336) vs. no PSU (545)	- Odds of being screened positive for disordered eating were higher in those who exceeded the smartphone addiction threshold (OR = 1.55).<br>- Odds of being screened positive for disordered eating were higher in people using smartphone for 7-8h/day (OR = 2.12) and >8h/day (OR = 2.73) but not 3-4 or 5-6h/day, compared with reference category (<2h/day).

Miranda et al., 2021 *Brazil*	Cross-sectional	School	405	100%	14-19 (15.9±1.3)	Smartphone screen time (min/day)	BSQ	-	- Adolescents with high screen time had higher body distortion compared with those with adequate screen time (OR = 1.59; *P*=.05).<br>- Body dissatisfaction was associated with a higher odds of having high screen time (OR = 1.5; *P*=.016).
Örnek and Gündoğmuş, 2022 *Turkey*	Cross-sectional	University	358	59.8%	NR (22.3±3.1)	SAS-SV	EAT-40	Smartphone addiction (183) vs. no smartphone addiction (175)	- Disordered eating scores were greater in those classed as smartphone addicted than those not classed as smartphone addicted (*P*<.001).<br>- In a linear regression model, smartphone addiction scores positively predicted EAT-40 scores when including age, gender, weight group, school year, age of smartphone and family income status in the model.
Park et al., 2022 *South Korea*	Cross-sectional	School	209	55.5%	NR (12.9±0.7)	Korean Smartphone Overdependence Scale for Adolescents	YFAS-C; CEBQ	High PSU risk (96) vs. no PSU risk (113)	- Smartphone overdependence scores were positively correlated with food addiction scores after adjusting for age, sex, BMI percentage and SES (r= 0.43; *P*<.001).<br>- Participants at high risk of PSU had greater food addiction scores and emotional overeating than participants at low risk of PSU.
Pekgör and Eryılmaz, 2021 *Turkey*	Cross-sectional	Hospital clinic	113	85.6%	18-65 (med, IQR: 35, 31-45)	SAS-SV	YFAS; CEBQ	Food addiction (38) vs. no food addiction (75)	- Rate of food addiction not higher in people with smartphone addiction compared to people without smartphone addiction (*P*=.076).<br>- Positive correlation between smartphone addiction score and food addiction score (r = 0.21; *P*=.033).
Peris et al., 2020 *Spain*	Cross-sectional	School	447	56.2%	13-16 (14.9±0.8)	ERA-RSI	BSS	-	- Nomophobia scores were not associated with body satisfaction scores (r = -0.03), although higher nomophobia scores were associated with higher self-perceived physical attractiveness (r = 0.13).<br>- Nomophobia was predicted by body satisfaction and physical attractiveness before and after adding gender, age, neuroticism, disinhibition and narcissism scores to the model.
Piko et al., 2022 *India*	Cross-sectional	High school	112	47.3%	14-18 (16.0±1.1)	SAS-SV	EAT-26	High risk for disordered eating (NR) vs. low risk (NR)	- Individuals at risk of disordered eating had higher smartphone addiction scores (OR = 1.07; *P*<.05).
Rozgonjuk et al., 2023 *Germany*	Cross-sectional	Community	119	100%	18-49 (23.1±4.6)	Daily screen time (h/day)	EDE-Q	Historical ED diagnosis (34) vs. no historical ED diagnosis (85)	- Smartphone use is positively associated with body dissatisfaction (r = 0.39) EDE-Q Global scores (r = 0.42) and all subscales other than the eating concern subscale (r range = 0.38-0.43)

									- Women with a history of an ED diagnosis spent significantly more time daily on their smartphones (*P*=.005).
Sanlier et al., 2024 *Turkey*	Cross-sectional	Community	643	62.7%	18-50 (26.5±9.6)	SAS	EAT-26	Normal internet use (460) vs. internet addict (183)	- Smartphone addiction scores were positively associated with EAT-26 scores (r = 0.16; *P*<.001).<br>- Scoring highly on the SAS was associated with a greater tendency to be classed as having an abnormal eating attitude (OR = 2.26; *P*<.001).
Sezer et al., 2025 *Turkey*	Cross-sectional	School	437	79.9%	15-26 (16.3±1.2)	SAS-SV	TFEQ	-	- Smartphone addiction scores were positively correlated with cognitive restraint (r = 0.28), emotional eating (r = 0.29) and uncontrolled eating (r = 0.33) subscales of the TFEQ.<br>- These associations were consistent in multiple regression analyses also including social media addiction scores and digital game addiction scores in the model.
Tayhan and Yabancı, 2021 *Turkey*	Cross-sectional	University	437	73.5%	19-29 (20.7±1.6)	SAS<br>Daily smartphone screen time (h/day)	EAT-40	-	- Participants categorised as high risk for an eating behaviour disorder (>30 on EAT-40) had higher smartphone addiction scores (*P*<.001) and higher daily smartphone screen time (*P*=.023).<br>- EAT-40 scores were positively correlated with social media addiction scores (r = 0.28; *P*<.001) and smartphone screen time (r = 0.14; *P*=.004).
Türkkan et al., 2025 *Turkey*	Cross-sectional	Community	604	82.9%	18-45 (21.1±3.0)	SAS-SV	TFEQ	-	- Positive correlation between smartphone addiction and uncontrolled eating (r = 0.40; *P*<.01), cognitive restriction (r = 0.21; *P*<.01) and emotional eating (r = 0.30; *P*<.01).<br>- The relationship between smartphone addiction and BMI was positively mediated by (higher) uncontrolled eating, cognitive restriction and emotional eating in three separate mediation models. However, the direct relationship between smartphone addiction and BMI was not significant.
Wang et al., 2023 *China*	Cross-sectional	University	1,112	61.1%	17-29 (21.4±3.2)	MPAI<br>Daily smartphone screen time (h/day)	EAT-26	Male (433) vs. female (678)	- EAT-26 scores were higher in those classed as smartphone addicted (*P*<.001).<br>- Significant positive correlation between EAT-26 scores and both smartphone addiction scores (r = 0.25; *P*<.001) and smartphone usage on the weekends (r = 0.09; *P*<.01) and weekdays (r = 0.10; *P*<.01).<br>- EAT-26 scores were positively associated with smartphone addiction scores when also including several other variables in a multiple regression model (e.g., BMI, depression and anxiety, smartphone usage, difficulty falling asleep at night, frequency of physical activity)

Watkins et al., 2025<br>*USA*	Cross-sectional	School	45	51.0%	11-14 (13.1±1.3)	Daily screen time (h/day)	BSQ, BAS-2	-	- Screen time, together with social media addiction scores and sex, was a positive predictor of BSQ scores (*P*<.001; Cohen's *d* for screen time = 0.39).
Wickord and Quaiser-Pohl, 2022<br>*Germany*	Cross-sectional	Community	398	78.2%	14-67 (25.9±11.1)	MPPUS-27	ISR	-	- PSU scores positively correlated with ED symptoms (r = 0.23; *P*<.01).<br>- Generation (digital immigrants >40 years and digital natives <40 years) moderated the relationship between ED symptoms and PSU, whereby the relationship between ED symptoms and PSU was stronger for digital immigrants than natives.
Wu et al., 2021<br>*China*	Cross-sectional	University	4,325	61.4%	NR (19.9±1.3)	SAS-SV	EAT-26	-	- PSU was positively associated with EAT-26 scores (r = 0.25; *P*<.001).<br>- The relationship between poor sleep quality and disordered eating behaviours was mediated by (higher) PSU.<br>- There was also serial mediation between sleep quality and disordered eating behaviours with (higher) PSU and (higher) psychological distress (both depression and anxiety) as serial mediators.
Yang et al., 2022<br>*China*	Cross-sectional	University	5,986	54.1%	17-32 (19.8±1.75)	SAS-SV	SAS<br>DEBQ	-	- Smartphone addiction scores were positively associated with body dissatisfaction (r = 0.25; *P*<.01), restrained eating (r = 0.37; *P*<.01), emotional eating (r = 0.39; *P*<.01) and external eating (r = 0.47; *P*<.01).<br>- Smartphone addiction scores mediated the relationship between body dissatisfaction and restrained eating, emotional eating and external eating.<br>- Sequential effects were also found with smartphone addiction scores à depression as sequential mediators.
Yang et al., 2020<br>*Singapore*	Cross-sectional	Community	100	100%	13-18 (15.1±1.3)	Daily smartphone screen time (h/day)	BES-21	-	After controlling for age, income, BMI and internal locus of control over one's body:<br>- The relationship between excessive smartphone use (>4 h/day) and poorer body esteem was sequentially mediated by (greater) cognitive internalisation of an ideal body image, (greater) appearance comparison, (greater) and appearance anxiety.<br>- This mediation model was no longer significant when controlling for total social media screen time, suggesting that smartphone usage without social media usage doesn't fortify cognitive internalisation of thin ideals.<br>- When controlling for social media use, browsing website, listening to music and watching TV shows were indirectly

									related to poorer body esteem via the aforementioned mediators

Abbreviations: APU = Addictive Patterns of Use Scale; ß = beta coefficient; BAS-2 = Body Appreciation Scale-2; BDD-Q = Body Dysmorphic Disorder Questionnaire; BDS = Body Dissatisfaction Scale; BED = Binge Eating Disorder; BES = Body Esteem Scale; BIAS-BD = Body Image Assessment Scale-Body Dimension; BIDS = Body Image Dissatisfaction Scale; BMI = Body Mass Index; BPSS = The Satisfaction and Dissatisfaction with Body Parts Scale; BSQ = Body Shape Questionnaire; BSS = Body Self-esteem Scale; C-EDE-QS = Chinese version of Short Form of the Eating Disorder Examination Questionnaire; CEBQ = Child Eating Behaviour Questionnaire; DEBQ = Dutch Eating Behaviour Questionnaire; EAT = Eating Attitudes Test; ED = eating disorder; EDE-Q = Eating Disorder Examination-Questionnaire; ERA-RSI = Scale of Risk of Addiction to Social Media and the Internet for Adolescents; h/day = hours per day; ISR = ICD-10 Symptom Rating Scale; KSADS-5 = Kiddie Schedule for Affective Disorders and Schizophrenia; M = mean; MBSRQ-AS = Multidimensional Body-Self Relations Questionnaire-Appearance Scale; MIDI = Minnesota Impulse Disorder Interview; MPAI = Mobile Phone Addiction Index; MPATS = Mobile Phone Addiction Tendency Scale; MPIQ = Mobile Phone Involvement Questionnaire; MPPUS-27 = Mobile Phone Problematic Use Scale; N = number; NMP-Q = Nomophobia Questionnaire; NR = not reported; OR = odds ratio; PSU = problematic smartphone use; PU = Problematic Mobile Phone Use Scale; r = correlation coefficient; SAS = Smartphone Addiction Scale; SAS-SV = Smartphone Addiction Scale Short Version; SCOFF = Sick Control One Fat Food; SD = standard deviation; SES = socio-economic status; SPAI-I = Italian version of the Smartphone addiction Inventory; T1 = timepoint 1; T2 = timepoint 2; TFEQ = Three-Factor Eating Questionnaire; YFAS = Yale Food Addiction Scale; YFAS-C = Yale Food Addiction Scale for Children.
